# Supplementary figures and images for: Circadian rhythms in sexual behavior and their influence on reproductive outcomes in mice
Source: bioRxiv. 2025 Nov 6:2025.11.05.686629. Preprint. [Version 1] doi: 10.1101/2025.11.05.686629 (PMC12637587; doi:10.1101/2025.11.05.686629)

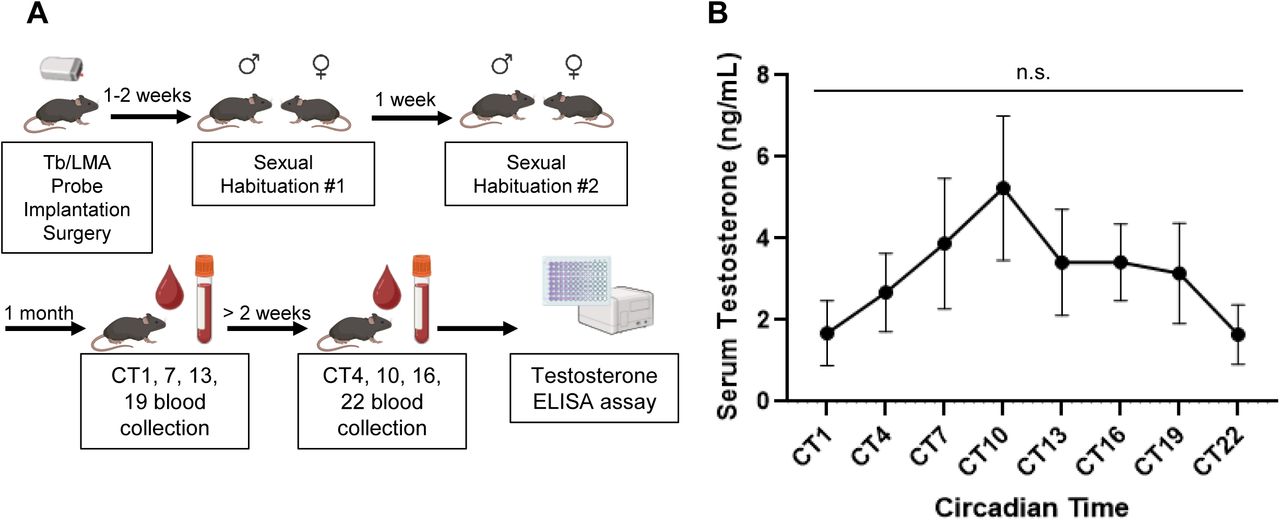

Supplement: figure-S1 — Supplementary Figure 1: Assessing testosterone levels across the circadian day. A) Male mice were implanted with body temperature/locomotor activity abdominal probes. After a 1-2 week recovery from surgery, they underwent their first overnight sexual habituation with an OVX, hormone-primed female. The second overnight sexual habituation occurred about 1 week later. At least 2 weeks after the second sexual habituation, male mice were dark adapted for at least 2 weeks. After being in DD for at least 2 weeks, blood collections began. Blood was collected from the tail vein every 30 hr beginning at CT1 and continuing at CT7, CT13, and CT19. After the CT19 blood draw, mice were returned to LD for at least 2 weeks before being returned to DD for CT4, CT10, CT16, and CT22 blood sampling to begin. Plasma from the blood collections was then used for a testosterone ELISA assay. B) Testosterone peaked at CT10, although significance was not reached. n.s.: not significant. N = 7-10 male mice per timepoint. [file figure-S1.jpg]
